# Experimental Chagas disease-induced perturbations of the fecal microbiome and metabolome

**DOI:** 10.1371/journal.pntd.0006344

**Published:** 2018-03-12

**Authors:** Laura-Isobel McCall, Anupriya Tripathi, Fernando Vargas, Rob Knight, Pieter C. Dorrestein, Jair L. Siqueira-Neto

**Affiliations:** 1 Skaggs School of Pharmacy and Pharmaceutical Sciences, University of California San Diego, La Jolla, California, United States of America; 2 Division of Biological Sciences, University of California San Diego, La Jolla, California, United States of America; 3 Department of Pediatrics, University of California San Diego, La Jolla, California, United States of America; 4 Center for Microbiome Innovation, University of California San Diego, La Jolla, California, United States of America; 5 Collaborative Mass Spectrometry Innovation Center, University of California San Diego, La Jolla, California, United States of America; 6 Department of Computer Science and Engineering, University of California San Diego, La Jolla, California, United States of America; Universidad del Valle de Guatemala, GUATEMALA

## Abstract

*Trypanosoma cruzi* parasites are the causative agents of Chagas disease. These parasites infect cardiac and gastrointestinal tissues, leading to local inflammation and tissue damage. Digestive Chagas disease is associated with perturbations in food absorption, intestinal traffic and defecation. However, the impact of *T*. *cruzi* infection on the gut microbiota and metabolome have yet to be characterized. In this study, we applied mass spectrometry-based metabolomics and 16S rRNA sequencing to profile infection-associated alterations in fecal bacterial composition and fecal metabolome through the acute-stage and into the chronic stage of infection, in a murine model of Chagas disease. We observed joint microbial and chemical perturbations associated with *T*. *cruzi* infection. These included alterations in conjugated linoleic acid (CLA) derivatives and in specific members of families *Ruminococcaceae* and *Lachnospiraceae*, as well as alterations in secondary bile acids and members of order Clostridiales. These results highlight the importance of multi-‘omics’ and poly-microbial studies in understanding parasitic diseases in general, and Chagas disease in particular.

## Author summary

Host-parasite interactions are usually studied as a binary system, without considering the role of the host microbiota. This work integrates microbiome research into the study of gastrointestinal Chagas disease. We show that *T*. *cruzi* infection perturbs the fecal microbiome and metabolome, indicating functional changes affecting the gastrointestinal lumen. Our results support further investigation into the role of the microbiota-parasite interaction in gastrointestinal Chagas disease pathogenesis.

## Introduction

*Trypanosoma cruzi* are protozoan parasites endemic to Central and South America. They cause a range of cardiac and gastrointestinal manifestations collectively known as Chagas disease. With increasing travel and immigration, infected individuals are also now found worldwide. Six to seven million people are *T*. *cruzi-*positive, thirty to forty percent of which will develop symptomatic disease decades after their initial exposure to the parasite. Cardiac symptoms are the most common; these include conduction abnormalities, arrhythmias, aneurysms, and heart failure leading to death. Clinically apparent gastrointestinal Chagas disease is less prevalent; gastrointestinal Chagas disease is associated with enlargement of the esophagus and/or colon (megaesophagus, megacolon), leading to pain, dysphagia, altered intestinal transit, altered nutrient intake, and constipation [1].

Research on cardiac Chagas disease progression has focused mainly on heart tissue. However, studies in murine models using luminescent *T*. *cruzi* cell lines showed recirculation of parasites from gastrointestinal tissues to the heart and propose a model in which gastrointestinal sites function as a reservoir for parasites to re-invade heart tissue and cause cardiac damage [2, 3]. These suggest an important role for intestinal *T*. *cruzi* infection beyond megasyndrome pathogenesis. Gastrointestinal sites may also be a major source of parasites during post-treatment recrudescence [4].

Gastrointestinal Chagas disease has a strong geographic association; most cases represent infections acquired in Bolivia, Brazil, Argentina and Chile. Disease tropism has been strongly tied to *T*. *cruzi* strain [5], but diet may also play a role [6]. *T*. *cruzi* infection is associated with parasite dose-dependent recruitment of inflammatory cells to the colon and colon damage [7], all of which could perturb the intestinal microbiota. Conflicting results comparing infection outcomes in germ-free and conventional mice have been reported, with one study showing similar survival [8], and another study showing differential survival [9]. The impact of *T*. *cruzi* infection on the gut microbiome and metabolome composition in immunocompetent animals has yet to be assessed. Such a system is more representative of human infection than germ-free models that show significant immunological defects [10]. This work applies 16S amplicon sequencing and mass spectrometry-based metabolomics on fecal pellets to characterize the functional bacterial changes associated with *T*. *cruzi* infection, in an immunocompetent murine model of Chagas disease. This joint approach enabled us to identify correlated microbiome and metabolome changes, and paves the way for further investigation of the *T*. *cruzi-*microbiota interaction in the context of Chagas disease pathogenesis.

## Methods

### Ethics statement

All vertebrate animal studies were performed in accordance with the USDA Animal Welfare Act and the Guide for the Care and Use of Laboratory Animals of the National Institutes of Health. Euthanasia was performed by isoflurane overdose followed by cervical dislocation. The protocol was approved by the University of California San Diego Institutional Animal Care and Use Committee (protocol S14187).

### *In vivo* experimental sample and data collection

Male C3H/HeJ mice were purchased from Jackson laboratories and allowed to acclimatize to our vivarium for 2 weeks before the start of experimentation. At day 0, mice were infected by intraperitoneal injection with 1,000 red-shifted luciferase-expressing *T*. *cruzi* strain CL Brener culture-derived trypomastigotes [2] (20 mice across four cages) or left uninfected (injected with DMEM media only, 20 mice divided in four cages), and initial fecal pellets collected. Parasite burden was measured bi-weekly during the acute stage of infection by bioluminescence imaging following D-luciferin injection using an In vivo Imaging System (IVIS) Lumina LT Series III (Perkin Elmer). Total body luminescence, cardiac region luminescence, and abdominal luminescence were determined using Living Image 4.5 software. Fecal pellets were collected by monitoring the mice until they defecated naturally, at which point the freshly excreted pellets were immediately collected and snap-frozen in liquid nitrogen. Fecal pellets were collected bi-weekly in the acute stage of disease; imaging and fecal collection were performed every 2–3 weeks during the chronic stage of disease. Each time point was analyzed individually; no samples were pooled.

No visual changes were observed at any time point for fecal pellets from infected mice compared to fecal pellets from uninfected mice. Infected mice showed no overt disease symptoms except slight decrease in weight at the last two collection timepoints (days 64 and 90, p<0.05, Mann-Whitney, FDR-corrected) (**S1A Fig**), although four mice were found dead over the course of the experiment (days 20, 63, 79 and 90 post-infection) (**S1B Fig**). Hematoxylin-eosin (H&E) staining of colon samples did not show any apparent tissue damage or inflammatory infiltrate in infected mice compared to uninfected mice (**S1D Fig**). However, parasite distribution through the gastrointestinal tract is highly localized during chronic stage of infection with luminescent CL Brener [2], and we cannot rule out the possibility that other colon regions were altered by infection.

### UHPLC-MS/MS analysis

Weighed fecal pellets were homogenized in 50% methanol spiked with 2 μM sulfachloropyridazine using a Qiagen TissueLyzer at 25 Hz for 5 min [11], at a constant concentration of 50 mg feces / 1000 μL of extraction solvent, followed by overnight incubation at 4°C. Samples were then centrifuged at 16,000g for 10 min. Equal volumes of centrifugation supernatant were dried in a vacuum concentrator and frozen at -80°C. For LC-MS/MS analysis, samples were resuspended in 50% methanol spiked with 2 μM sulfadimethoxine and analyzed on a Maxis Impact HD QTOF mass spectrometer (Bruker Daltonics) coupled to an UltiMate 3000 UHPLC system (Thermo Scientific). A given infected or uninfected mouse was randomly assigned to one of eight 96 well plates, alternating infected and uninfected samples. Time-course samples were plated left to right in the 96 well plates, while run order was top to bottom. Controls included blanks (resuspension solvent) and pooled QC controls every 16 samples, and a standard mix of six compounds (sulfamethazine, sulfamethizide, sulfachloropyridazine, sulfadimethoxine, amitryptiline and coumarin) with known retention time at the beginning of the run and between each plate, to monitor for retention time shifts.

Liquid chromatography separation was performed on a 1.7 μm C18 (50 × 2.1 mm) UHPLC column (Phenomenex) heated to 40°C, with water + 0.1% formic acid as mobile phase A and acetonitrile + 0.1% formic acid as mobile phase B, at a constant flow rate of 0.5 mL/min. The LC gradient was: 0–1 min, 5% B; 1–9 min linear ramp up to 100% B; 9–11 min hold at 100% B; 11–11.5 min ramp down to 5% B; 11.5–12.5 min hold at 5% B.

Ions were generated by electrospray ionization and MS spectra acquired in positive ion mode with the following instrument parameters: nebulizer gas pressure, 2 Bar; Capillary voltage, 3,500 V; ion source temperature, 200°C; dry gas flow, 9.0 L/min; spectra rate acquisition, 3 spectra/s. MS/MS data was collected by fragmentation of the five most intense ions, in mass range 50–1,500 *m/z*, with active exclusion after 2 spectra and release after 30s. Mass ranges representing common contaminants and the lock masses were also excluded (exclusion list 144.49–145.49, 621.00–624.10, 643.80–646.00, 659.78–662.00, 921.00–925.00, 943.80–946.00, 959.80–962.00). Ramped collision-induced dissociation energy parameters ranged from 10–50 eV. Daily calibration was performed with ESI-L Low Concentration Tuning Mix (Agilent Technologies). Hexakis(1H,1H,3H-tetrafluoropropoxy)phosphazene (Synquest Laboratories), m/z 922.009798, was present throughout the run and used as internal calibrant (lock mass).

### UHPLC-MS/MS data analysis

LC-MS/MS raw data files were lock mass-corrected and converted to mzxml format using Compass Data analysis software (Bruker Daltonics). MS1 feature identification was performed using an OpenMS-based [12] workflow (Optimus version 1.1.0 https://github.com/alexandrovteam/Optimus, see **S1 Table** for parameters), restricting to features with MS2 data available. Feature abundance was normalized to the sulfachloropyridazine extraction control. Principle coordinates analysis (PCoA) was performed on the normalized data with our in-house tool ClusterApp using the Bray-Curtis-Faith dissimilarity metric [13, 14], and visualized in EMPeror [15]. Molecular networking was performed on the Global Natural Products Social Molecular Networking platform (GNPS) [16], with the following parameters: parent mass tolerance 0.02 Da, MS/MS fragment ion tolerance 0.02 Da, cosine score 0.6 or greater, at least 4 matched peaks, maximum analog mass shift, 200 Da. Molecular networks and correlation networks were visualized with Cytoscape 3.4.0 [17]. Most metabolites were identified to levels 2/3 according to the 2007 metabolomics standards initiative (putatively annotated compounds or compound classes [18]). Additional putative annotations were performed using the LIPID MAPS *m/z* search tool [19]. Linoleic acid/conjugated linoleic acid (LA/CLA) were identified with higher confidence by retention time and spectral matching to authentic standards (Spectrum Chemical/Sigma Aldrich; level one annotation [18]). Random forest analysis over 5,000 trees was performed in R [20].

### 16S rRNA sequencing

DNA extraction, 16S library preparation and sequencing were performed according to standard protocols from the Earth Microbiome project (http://www.earthmicrobiome.org/protocols-and-standards/ [21]). Briefly, DNA extraction was performed using the MO BIO PowerSoil DNA Isolation Kit (MoBio Laboratories). PCR amplification targeting the V4 region of the 16S rRNA bacterial gene was performed with barcoded primers 515F/806R as described in [22]. Equal amounts of amplicons from each sample were pooled in equal concentration and cleaned with the MoBio UltraClean PCR Clean-Up Kit. Library was PhiX-spiked and sequenced on the UC San Diego Institute for Genomic Medicine Illumina MiSeq2000 platform.

### 16S marker gene data analysis and joint microbiome-metabolome analyses

Raw FASTQ data files were demultiplexed using Qiita (https://qiita.ucsd.edu, study ID 10767) with the following parameters: maximum barcode errors: 1.5; sequence maximal ambiguous bases: 0; maximal bad run length: 3; Phred quality threshold: 3. This resulted in 12,307,767 high-quality reads with a median of 24,578 sequences per non-blank sample. Closed-reference Operational Taxonomic Unit (OTU) picking was performed in Qiita with 97% sequence identity using sortmeRNA [23] as the clustering algorithm. Subsequent data analysis was performed using the QIIME1 pipeline [24], rarefying to 8,500 reads per sample. PCoA plots were generated using the weighted UniFrac distance metric [25] and visualized in EMPeror [15]. Random forest analysis over 5,000 trees [20] was performed in R using jupyter notebooks [26].

Procrustes analyses [27, 28] were performed using the QIIME1 [24] scripts *beta_diversity*.*py* to generate the weighted UniFrac distance matrix (16S data) or Bray-Curtis-Faith distance matrix (LC-MS data), followed by *principal_coordinates*.*py* to perform principal coordinates analysis. PCoA outputs were used as input for *transform_coordinate_matrices*.*py* (Procrustes), with 1000 random permutations. The output of this analysis was visualized EMPeror [15].

Groups of bacteria and metabolites correlated with infection status were identified by Weighted Correlation Network Analysis (WGCNA) analysis. Average hierarchical clustering using the WGCNA R package in combination with soft-thresholded Pearson correlation was performed to independently cluster highly correlated microbes and metabolites into modules [29]. Data was pre-filtered using the *goodSampleGenes* function of the WGCNA package to remove metabolites or OTUs with >50% missing values. Remaining outlier samples were removed using the *cutreeStatic* function, with a minimum size of 10. Soft thresholding power was determined using the *pickSoftThreshold* function and set to 4 for metabolites and for 30 microbiome data. Minimum module size was 30 for OTUs and 10 for metabolite features; threshold for merging modules was 0.25. Using this approach, 49 metabolite modules and three microbial modules were obtained. Microbial and chemical modules were independently correlated with parasite burden using Pearson correlation. Since we were interested in identifying the changes in gut ecosystem due to parasite infection specifically, only the modules that were significantly correlated with parasite burden were retained for downstream analysis (Student asymptotic p-value <0.01; positive correlation coefficient). This represented nine metabolite modules and one microbial module. We performed pairwise Pearson correlation between these modules, which yielded six positively correlated microbe-metabolite module pairs (Student asymptotic p-value <0.01). Finally, we performed pairwise Pearson correlations between microbial and chemical components of these strongly correlated module pairs to obtain candidate microbial-metabolite associations relevant to *T*. *cruzi* infection in mice (positive correlation, p<0.05). These metabolites were then compared with molecular networking results to identify common members of chemical families. Correlations between the members of these families and bacterial OTUs were plotted using Cytoscape 3.4.0 [17].

## Results and discussion

*T*. *cruzi* infection perturbs the fecal microbiome and metabolome

**Fig 1:**
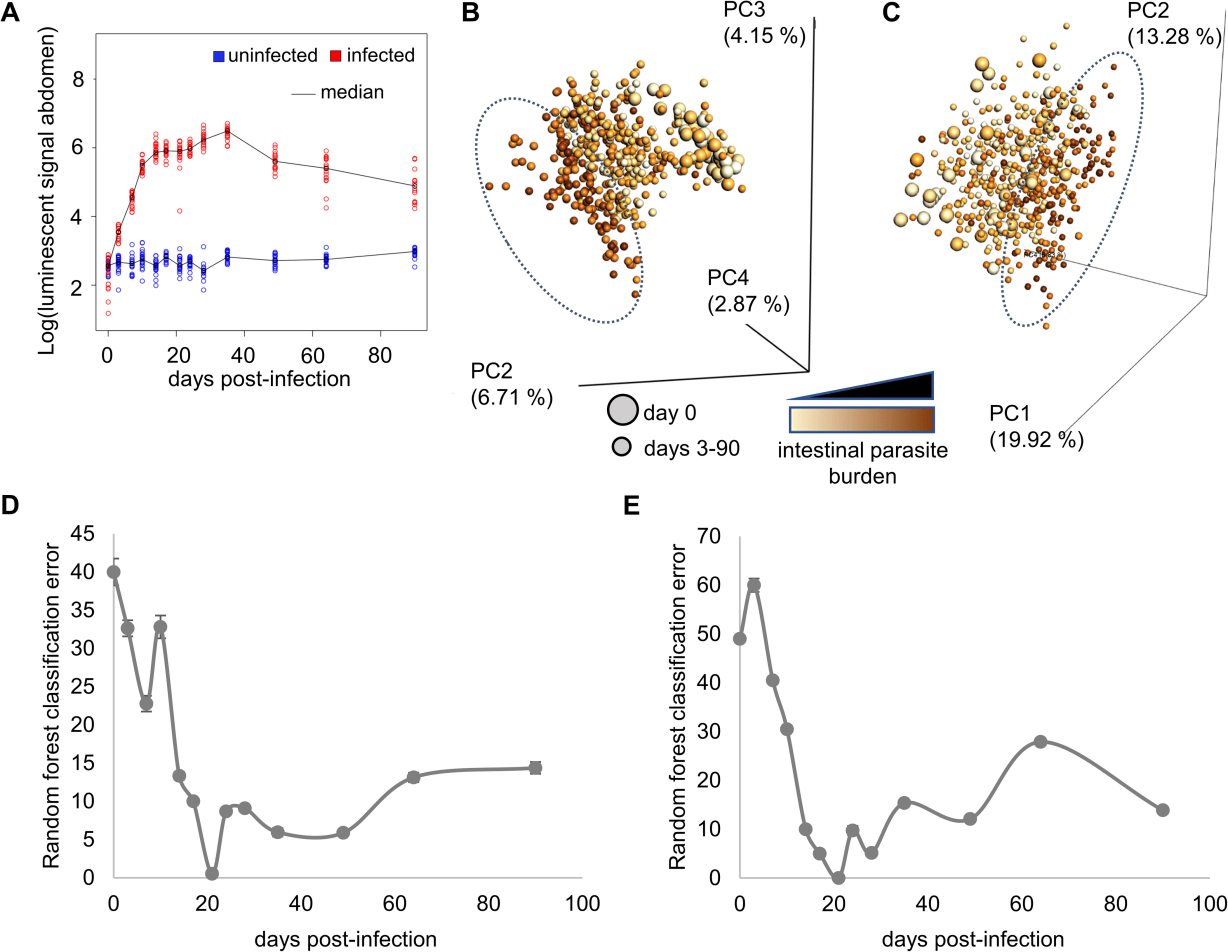
Infection with *T*. *cruzi* perturbs the fecal microbiome and metabolome. (**A**) Intestinal parasite burden progression during the acute and chronic stage of the disease. Parasite signal peaked at day 35 post-infection and decreased into the chronic stage of the disease. (**B, C**) Principal coordinates analysis (PCoA) showing clustering of high parasite burden samples (dotted oval) compared to uninfected and low parasite burden samples for fecal microbiome (**B**, weighted UniFrac distance metric) and fecal metabolome (**C**, Bray-Curtis-Faith distance metric). Each sphere represents a single sample from one mouse at a given timepoint. Spheres are colored by intestinal parasite burden, with darkest spheres coming from samples collected at the peak of infection, when parasite burden is highest. Samples collected prior to infection are shown by large spheres, with all other samples represented by small spheres. (**D,E**) Random forest classification error between infected and uninfected samples using microbiome (**D**) and metabolome (**E**) datasets. Classifier is unable to distinguish between infected and uninfected samples initially. Classification accuracy improves over the acute stage of infection, with near-perfect classification 21 days post-infection. Error then increases during the chronic disease stage, although classification accuracy remains better than pre-infection.

To determine the impact of *T*. *cruzi* infection on the fecal metabolome and microbiome, we followed C3H/HeJ mice infected with a bioluminescent *T*. *cruzi* strain for 3 months post-infection. In this system, abdominal parasite burden peaked at 35 days post-infection (**Fig 1A**). Fecal samples were collected twice a week during the acute stage and every 2–3 weeks during the chronic stage of disease. Fecal bacterial operational taxonomic units (OTUs) were identified by sequencing of the V4 hypervariable region of the 16S rRNA genes [30]. Untargeted mass spectrometric analysis of the collected fecal pellets was performed by liquid chromatography-mass spectrometry followed by molecular networking for metabolite identification [16]. Detected identifiable metabolites include known products specifically from gastrointestinal microbes (secondary bile acids, tryptophan metabolites…), metabolites that can be found in the diet and/or modified by gut microbes (*e*.*g*. conjugated linoleic acid and derivatives generated from dietary linoleic acid) as well as common host and microbial metabolites (amino acids, phospholipids…). Overall bacterial composition was strongly affected by parasite burden (**Fig 1B**), as was the overall fecal metabolome (**Fig 1C**). Interestingly, infected to uninfected average distances reached their maximum before peak parasite burden (**S2 Fig**), with the best discriminatory ability between infected and uninfected samples at day 21 post-infection for both metabolome and microbiome (**Fig 1D and 1E**).

### Synchronized changes in the fecal microbiome and metabolome during experimental Chagas disease

**Fig 2:**
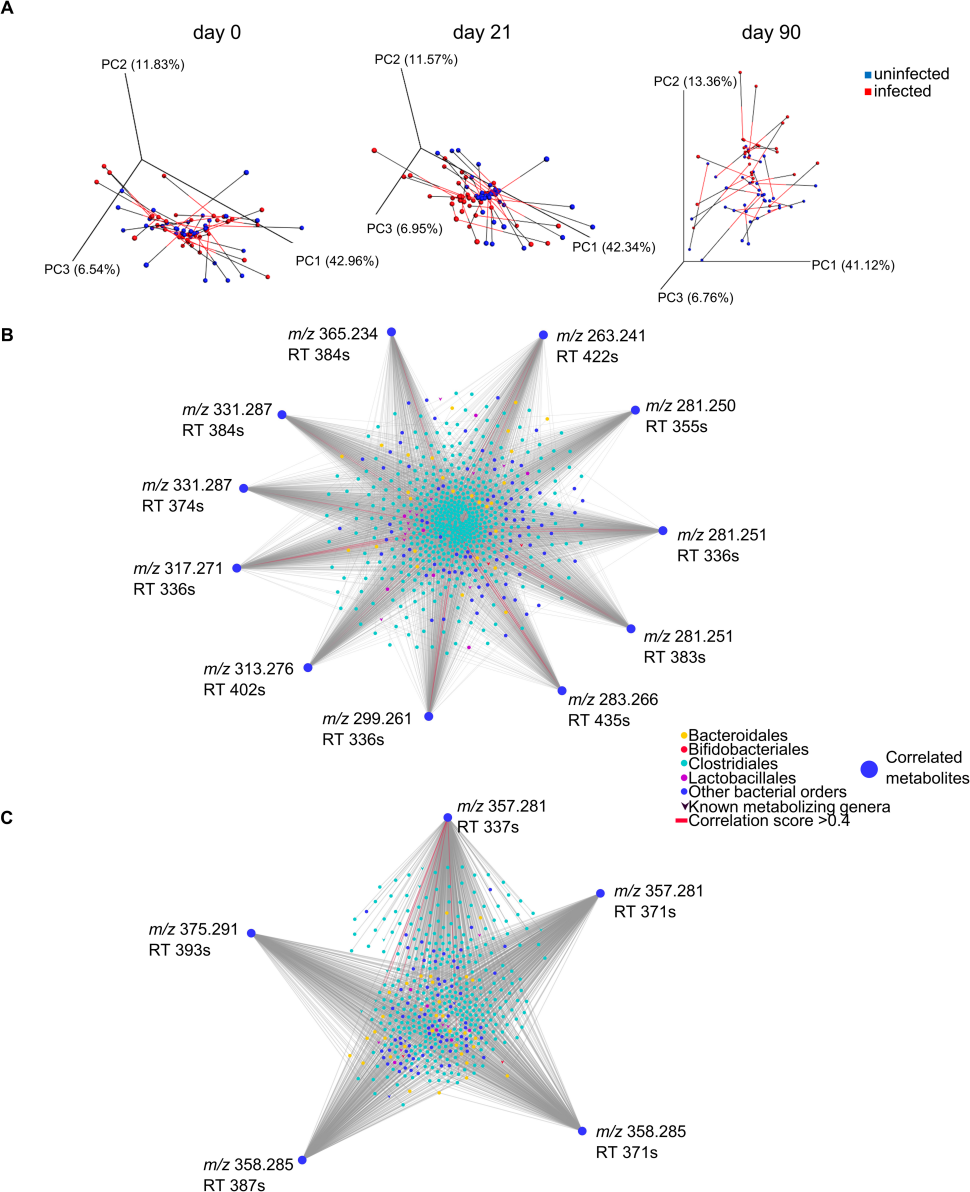
Joint microbial and chemical alterations during experimental Chagas disease. (**A**) Procrustes analysis of microbiome and metabolome data, showing similar overall trends for microbiome and metabolome: lack of segregation between infected and uninfected samples at day 0, and clear separation at day 21. Less separation was observed 90 days post-infection. Connected spheres came from the same sample. Black lines indicate metabolome data and red lines microbiome data. (**B**) Network of correlated CLA derivatives (outer perimeter) and bacterial OTUs (central circles and V shapes), as identified by WGCNA analysis (correlation coefficients > 0, p-value <0.05). Known metabolizing bacterial genera are V-shaped, all other OTUs are represented by circles. OTU nodes are colored based on corresponding bacterial order, to highlight members of the Bacteroidales, Bifidobacteriales, Clostridiales, Lactobacillales orders. (**C**) Network of correlated cholic acid derivatives and bacterial OTUs, as identified by WGCNA analysis (correlation coefficients > 0, p-value <0.05). Known metabolizing bacterial genera are V-shaped, all other OTUs are represented by circles. OTU nodes are colored based on corresponding bacterial order.

Gut microenvironments are influenced by dietary components and by bacterial and host metabolism, all of which could affect parasite nutritional availability and antiparasitic immune responses [31]. Likewise, chemical changes in the gut microenvironment would influence bacterial growth and composition [32]. We therefore investigated the integration between the microbial and chemical changes we observed during experimental *T*. *cruzi* infection by performing Procrustes analysis [27, 28]. Separation between infected and uninfected fecal microbiome and metabolome samples jointly was observed at days 21 and 90 post-infection but not at day 0 (**Fig 2A, S2 Table**).

To determine the nature of these joint changes, we performed weighted gene co-expression network analysis (WGCNA) [29, 33] on microbial and chemical data. Metabolites and microbes were individually clustered into modules, and microbial and chemical modules correlated with abdominal parasite burden (abdominal luminescence) were identified (significance cutoffs: Student asymptotic p-value <0.01; correlation coefficients > 0). Only one module of 1954 bacterial OTUs (out of three bacterial modules) was correlated with parasite burden (Pearson correlation coefficient, 0.19; Student asymptotic p-value, 3e-05), **S3 Fig**). Nine metabolite modules (out of 49 metabolite modules) were correlated with parasite burden (Student asymptotic p-value <0.01, Pearson correlation coefficient 0.13–0.33, **S4 Fig**). Pair-wise correlation was then performed between these burden-correlated microbial and chemical modules, six of which showed statistically significant correlation (Student asymptotic p-value<0.01, Pearson correlation coefficient 0.13–0.52, **S5 Fig**). Metabolite feature to OTU pair-wise comparisons were then performed within each metabolite-microbe module pair (cutoffs: positive correlation, p<0.05).

Within these six correlated module pairs, almost all the metabolite features positively correlated with parasite burden were from different molecular subnetworks, suggesting that they are part of different chemical families [16]. Strikingly however, eleven metabolite features from the most strongly correlated metabolite module (Pearson correlation coefficient 0.33, p-value, 3e-13) were from the same molecular subnetwork of linoleic acid derivatives (**Table 1, S6A and S7 Figs**). Dietary linoleic acid (LA) is modified in the gut environment by bacteria from the genera *Lactobacillus*, *Bifidobacterium* and *Enterococcus* into conjugated linoleic acid (CLA) and further derivatives [34, 35]. Conjugated linoleic acid can also be taken up in the diet and further modified in the gastrointestinal tract [34–36]. *m/z* 281.251 RT 485s was confirmed as LA or CLA by retention time and accurate mass matching to authentic LA/CLA standards (level one annotation according to the 2007 metabolomics standards initiative [18]; **S6B Fig**). Our chromatography conditions do not enable clear differentiation of LA and CLA. Specific members of the orders Bacteroidales and Clostridiales, including members of the families *Ruminococcaceae* and *Lachnospiraceae* can hydrogenate CLA [37, 38], and indeed we observed the strongest correlation (Pearson correlation coefficient >0.4) between members of the order Clostridiales and *m/z* 283.266 RT 435s, putatively identified as vaccenic acid (**Fig 2B, S3 Table**). Microbial hydration of linoleic acid by members of the *Pediococcus* and *Lactobacillus* genera has also been reported [35, 37, 39–41]. Molecular networking indicates that *m/z* 299.261 RT 336s and *m/z* 317.271 RT 336s could represent single and double hydration products of linoleic or conjugated linoleic acid; they were correlated with specific *Ruminococcaceae* and *Lachnospiraceae* family members (**Fig 2B, S3 Table**). CLA absorption in the colon is limited; bacterial metabolites of linoleic acid therefore primarily exert their effects locally [42]. Linoleic acid metabolism products alter gut inflammatory responses, by promoting regulatory T cell recruitment [43], decreasing TNF receptor expression [39] and TNFα production [44], and increasing the anti-inflammatory cytokine TGFβ in the colon [44]. These metabolites could therefore promote gut microenvironments favoring *T*. *cruzi* persistence and gastrointestinal reservoir function.

**Table 1:** Conjugated linoleic acid-related molecules correlated to parasite burden.

*m/z*	Retention time (sec)	Mass difference to CLA	Putative identification	Known bacterial producers	Correlated bacteria from known producing families	Ref.
263.241	422	-18	-	-	-	-
281.250	355	0 (isomer 2)	LA/CLA isomer	-	-	-
281.251	336	0 (isomer 1)	LA/CLA isomer	-	-	-
281.251	383	0 (isomer 3)	LA/CLA isomer	-	-	-
283.266	435	+2	vaccenic acid	Order Bacteroidales, Clostridiales (families *Ruminococcaceae* and *Lachnospiraceae*)	Several members of order Clostridiales	[37, 38]
299.261	336	+18	LA/CLA single hydration product	*Pediococcus* and *Lactobacillus* genera	*Ruminococcaceae* and *Lachnospiraceae* family members	[35, 37, 39–41]
313.276	402	+32	-	-	-	-
317.271	336	+36	LA/CLA double hydration product	*Pediococcus* and *Lactobacillus* genera	*Ruminococcaceae* and *Lachnospiraceae* family members	[35, 37, 39–41]
331.287	374	+50	-	-	-	-
331.287	384	+50	-	-	-	-
365.234	384	+84	-	-	-	-

An additional group of 5 co-modulated features networked with cholic acid (**Table 2, S8 Fig**). *m/z* 357.281 RT 337s, *m/z* 357.281 RT 371s and *m/z* 375.291 RT 393s are identified as different close isomers or adducts of deoxycholic acid (level two annotation according to the 2007 metabolomics standards initiative [18]). Host-produced primary bile acids such as cholic acid are conjugated to taurine or glycine in the liver. Further modifications of primary bile salts are specifically performed in the gastrointestinal environment: members of the gut microbiota deconjugate primary bile salts and remove the 7-hydroxy group to form secondary bile acids such as deoxycholic acid. *Bacteroides*, *Bifidobacterium*, *Clostridium*, *Lactobacillus* and *Listeria* genera deconjugate bile acids, which are then dehydroxylated by *Clostridium* and *Eubacterium* genera [45]. Indeed, one member of the *Clostridium* genus, *Clostridium celatum* (OTU ID 4315688) was correlated with *m/z* 357.281 RT 337s (Pearson correlation coefficient 0.21028, p-value = 0.00000426), and weakly correlated with *m/z* 357.281 RT 371s and *m/z* 358.285 RT 371s (respective correlation coefficient, 0.09079 and 0.09770; respective p-values, 0.049161208 and 0.034207271) (**S4 Table**). Likewise, two members of the *Bifidobacterium* genus were correlated with *m/z* 357.281 RT 371s and *m/z* 375.291 RT 393s, and five members of the *Lactobacillus* genus were correlated with *m/z* 357.281 RT 371s, *m/z* 358.285 RT 371s and *m/z* 375.291 RT 393s (**S4 Table**). Further modifications can be performed by these genera and by *Escherichia*, *Egghertella*, *Fusobacterium*, *Peptococcus*, *Peptostreptococcus*, *Ruminococcus* genera [45], several of which were also correlated with our infection-modulated secondary bile acids (**Fig 2C**). The OTUs most strongly correlated with these secondary bile acids in our experiment (correlation coefficient >0.4) were also members of the order Clostridiales, either from the genus *Oscillospira* or from unidentified genera (**S4 Table**). Bile acid metabolism by the gut microbiota has been tied to local colon inflammation and general health [45], all of which could affect Chagas disease pathogenesis.

**Table 2:** Cholic acid/deoxycholic acid-related molecules correlated with parasite burden.

*m/z*	Retention time (sec)	Putative identification	Correlated bacteria examples
357.281	337	deoxycholic acid ([M+H-2H_2_O]), or close isomer	*Clostridium celatum*, order Clostridiales members
357.281	371	deoxycholic acid ([M+H-2H_2_O]), or close isomer	*Clostridium celatum*, *Bifidobacterium* spp., *Lactobacillus* spp.
358.285	371	-	*Clostridium celatum*, *Lactobacillus* spp.
358.285	387	-	-
375.291	393	deoxycholic acid ([M+H-H_2_O])	*Bifidobacterium* spp., *Lactobacillus* spp.

Several of these microbiome changes have been associated with other gastrointestinal diseases. *Lactobacillus* genus in particular is increased in obese individuals, while genus *Bifidobacterium* is decreased [46]. Members of the *Lactobacillus* genus and some *Bifidobacterium* species are increased in ileal Crohn’s disease, while members of order Clostridiales and family *Lachnospiraceae* are decreased [46]. Large-scale perturbations are also observed in these diseases, such as for example a trend for increased Firmicutes to Bacteroidetes ratio in obese individuals compared to lean individuals [46]. The observed microbial and metabolic perturbations in *T*. *cruzi-*infected animals may be a consequence of parasite-mediated modulations of local gastrointestinal microenvironments, such as nutrient depletion, or an off-target effect of anti-parasitic immune responses. Parasite control is associated with reactive oxygen and nitrogen species [47], which are known to affect the gut microbiome composition by killing bacterial species sensitive to oxidative stress while promoting the growth of species that use nitrate as a terminal electron acceptor for respiration [48]. Significant bacterial and metabolic changes become apparent by day 14 post-infection (**Figs 1D and 1E and S2**), which coincides with induction of adaptive immune responses to *T*. *cruzi* [49], suggesting an immune-mediated role in this disruption.

Given the anti-inflammatory roles of the hydrated linoleic acid metabolites we found altered by infection [39, 43, 44], the gut microbiome and metabolome changes we observed may be promoting long-term parasite gastrointestinal persistence and enabling the gastrointestinal tract to serve as a parasite reservoir. Microbiota perturbation may also contribute to the nutrient malabsorption and constipation observed in megasyndromes [50]. Modulating the infection-associated changes in the gut microbiome and its metabolism may prove to be an effective way to mitigate disease symptoms, nifurtimox gastrointestinal side effects or prevent parasite dissemination from the gastrointestinal tract to the heart. Modifying the levels of anti-inflammatory conjugated linoleic acid metabolites may be particularly useful in this context. Finally, although production of bile acid metabolites is performed in the gut environment by the local microbiota, these metabolites can be re-absorbed and circulate throughout the body, with far-ranging effects [51]. Bile acid metabolites may therefore also affect cardiac Chagas disease pathogenesis. Future work will directly investigate these possibilities, by testing whether the gut microbiome perturbations and the metabolites identified in this study are associated with Chagas disease severity, and assessing whether microbiome perturbation affects Chagas disease progression.

## Conclusions

Research on Chagas disease pathogenesis has focused on the interaction between the mammalian host and the parasite. Our results indicate that infection modulates the fecal microbiome, suggesting that host-microbe interaction research in the context of Chagas disease should also include the microbiota and not just *T*. *cruzi*. By integrating microbiome with metabolome data, we show that these microbial alterations are associated with functional changes in the gut chemical environment that could be affecting host inflammatory responses. These results support additional investigation into the *T*. *cruzi-*microbiota connection and into the role of the microbiota in Chagas disease pathogenesis. Given new evidence on the role of gastrointestinal persistence in parasite recrudescence [4], and our limited understanding of gastrointestinal Chagas disease compared to cardiac Chagas disease, such studies are essential to identify treatment strategies able to achieve sterile cure. Microbiota- and microbial metabolism-modulating therapies are now actively being developed for other cardiovascular diseases [52, 53]. Our results demonstrate that such approaches are likely to be beneficial in cardiovascular Chagas disease. Modulation of the gut microbiota or its metabolism may also be a promising strategy for megasyndrome patient management, or to slow progression of asymptomatic individuals to symptomatic disease.

## Supporting information

S1 Methods Supplemental methods and references. (DOCX)

S1 Table Optimus feature finding parameters. (DOCX)

S2 Table Statistics for procrustes analysis of microbiome and metabolome datasets with 1,000 Monte Carlo permutations. (DOCX)

S3 Table Pairwise correlation of parasite burden-correlated microbial OTUs and LA/CLA metabolite features. (CSV)

S4 Table Pairwise correlation of parasite burden-correlated microbial OTUs and secondary bile acid metabolite features. (CSV)

S1 Fig Disease progression. (**A**) Weight change. *, p<0.05 (Mann-Whitney, FDR-corrected). (**B**) Mortality. (**C**) Luminescence. (**D**) Representative hematoxylin-eosin staining of colon segments from uninfected (top) and infected (bottom) mice.
(DOCX)

S2 Fig Within-group and between-group distances. (**A**) Microbiome dataset (weighted UniFrac). (**B**) Metabolome dataset (Bray-Curtis-Faith). *, p<0.05 (Mann-Whitney, FDR-corrected).
(DOCX)

S3 Fig Microbial module correlation with abdominal parasite burden. Values in parentheses indicate Student asymptotic p-value for the correlation.
(DOCX)

S4 Fig Metabolite module correlation with abdominal parasite burden. Values in parentheses indicate Student asymptotic p-value for the correlation.
(DOCX)

S5 Fig Parasite burden-associated microbial and metabolite module correlation. Values in parentheses indicate Student asymptotic p-value for the correlation.
(DOCX)

S6 Fig Linoleic acid/conjugated linoleic acid identification. (**A**) LA/CLA Molecular network. (**B**) CLA isomers. Extracted ion chromatogram for *m/z* 281.200–281.260 (green). Numbers indicate CLA isomers with similar MS/MS fragmentation and arrow indicates sample peak matched to linoleic acid/conjugated linoleic acid authentic standard (red/blue). (**C**) Mirror plot showing spectral match of experimental spectrum (top, black) to library reference for CLA (bottom, green). (**D**) Overall comparable levels of LA/CLA between infected and uninfected samples. *, p<0.01 (Mann-Whitney, FDR-corrected).
(DOCX)

S7 Fig Co-modulated LA/CLA derivatives. (**A**) *m/z* 263.241 RT 422s. (**B**) *m/z* 281.250 RT 355s. (**C**) *m/z* 281.251 RT 336s. (**D**) *m/z* 281.251 RT 383s. (**E**) *m/z* 283.266 RT 435s. (**F**) *m/z* 299.261 RT 336s. (**G**) *m/z* 313.276 RT 402s. (**H**) *m/z* 317.271 RT 336s. (**I**) *m/z* 331.287 RT 374s. (**J**) *m/z* 331.287 RT 384s. (**K**) *m/z* 365.234 RT 384s. *, p<0.05 (Mann-Whitney, FDR-corrected).
(DOCX)

S8 Fig Co-modulated cholic acid derivatives. (**A**) Mirror plot showing spectral match of experimental spectrum (top) to cholic acid [M+H-3H_2_O] library reference (bottom). (**B**) Cholic acid molecular network. (**C**) Comparable cholic acid levels in infected and uninfected mice. (**D**) m/z 357.281 RT 337s. (E) m/z 357.281 RT 371s. (F) m/z 358.285 RT 371s. (**G**) m/z 358.285 RT 387s. (**H**) m/z 375.291 RT 393s. *, p<0.05 (Mann-Whitney, FDR-corrected).
(DOCX)

S9 Fig Pre-infection fecal microbiome composition. (**A**) Phylum level. (**B**) Class level. (**C**) Order level. (**D**) Family level. Each bar represents a given mouse.
(DOCX)

S10 Fig Principal coordinates analysis of metabolomics samples and controls. Various views and principal coordinates of PCoA plots with samples, blanks and pooled QC samples (**A**) and with only samples and pooled QC (**B**). Blank samples are distinctly different from all other samples, while pooled QC are in the middle of the PCoA. Distinct clustering of high parasite burden samples is highlighted with dotted oval, where permitted by the viewing angle.
(DOCX)
