# Supplementary material for: Experimental Chagas disease-induced perturbations of the fecal microbiome and metabolome
Source: PLoS Negl Trop Dis. 2018 Mar 12;12(3):e0006344. doi: 10.1371/journal.pntd.0006344 (PMC5864088; doi:10.1371/journal.pntd.0006344)
Supplement: S1 Methods — (DOCX) [file pntd.0006344.s001.docx]

**Supplemental methods**

Extracted ion chromatograms (S6 Fig) were generated using MZmine 2.21 [1].

For histology (**S1 Fig**), ~1 cm-long colon segments were fixed in formalin for 24h and progressively dehydrated with ethanol. Paraffin embedding, sectioning, and hematoxylin-eosin staining were performed at the UCSD Histology core. Slides were imaged on a NanoZoomer 2.0HT Slide Scanning System (Hamamatsu, Japan) at the UCSD Neuroscience Core.

For **S10 Fig** PCoAs: molecular networking was re-run with same parameters as in **Methods**, on samples and controls (data can be accessed at https://gnps.ucsd.edu/ProteoSAFe/status.jsp?task=207101872d2a4399b6c26bdce886c6e4). The GNPS output features table was normalized to total ion current. Principal coordinates analysis (Bray-Curtis-Faith metric) was performed in QIIME1 [2] and the output was visualized in Emperor [3].

**Supplemental references**

1. Pluskal T, Castillo S, Villar-Briones A, Orešič M. MZmine 2: modular framework for processing, visualizing, and analyzing mass spectrometry-based molecular profile data. Bmc Bioinformatics. 2010;11(1):395.

2. Caporaso JG, Kuczynski J, Stombaugh J, Bittinger K, Bushman FD, Costello EK, et al. QIIME allows analysis of high-throughput community sequencing data. Nat Methods. 2010;7(5):335-6. doi: 10.1038/nmeth.f.303. PubMed PMID: 20383131; PubMed Central PMCID: PMCPMC3156573.

3. Vazquez-Baeza Y, Pirrung M, Gonzalez A, Knight R. EMPeror: a tool for visualizing high-throughput microbial community data. Gigascience. 2013;2(1):16. doi: 10.1186/2047-217X-2-16. PubMed PMID: 24280061; PubMed Central PMCID: PMCPMC4076506.
