# Supplementary material for: Experimental Chagas disease-induced perturbations of the fecal microbiome and metabolome
Source: PLoS Negl Trop Dis. 2018 Mar 12;12(3):e0006344. doi: 10.1371/journal.pntd.0006344 (PMC5864088; doi:10.1371/journal.pntd.0006344)
Supplement: S1 Table — (DOCX) [file pntd.0006344.s002.docx]

**S1 Table. Optimus feature finding parameters.**

| **Parameter ^1^** | **Value** |
| --- | --- |
| **Detect LC-MS features** | |
| m/z tolerance (ppm) | 10.0 |
| Noise threshold | 2000 |
| MS2 feature precursor tolerance (Da) | 0.01 |
| MS2 feature retention time tolerance (s) | 20 |
| **Feature alignment and quantification** | |
| Retention time tolerance (s) | 20 |
| Reintegration of missing features | enabled |
| **Filter features** | |
| Minimum intensity ratio compared to blanks | 2.0 |
| **Match features by *m/z*-RT** | |
| Matched to feature table from GNPS |  |
| **Normalize features** | |
| Feature normalization | Enabled |
| Normalization using internal standards (sulfachloropyridazine extraction control) |  |

^1^ All other parameters were kept at default values
