# Supplementary material for: Experimental Chagas disease-induced perturbations of the fecal microbiome and metabolome
Source: PLoS Negl Trop Dis. 2018 Mar 12;12(3):e0006344. doi: 10.1371/journal.pntd.0006344 (PMC5864088; doi:10.1371/journal.pntd.0006344)
Supplement: S2 Table — (DOCX) [file pntd.0006344.s003.docx]

**S2 Table. Statistics for Procrustes analysis of microbiome and metabolome datasets with 1,000 Monte Carlo permutations.**

| **Day post-infection** | **M2** | **Monte-Carlo p-value** |
| --- | --- | --- |
| 0 | 0.875 | 0.01 |
| 21 | 0.907 | 0.061 |
| 90 | 0.775 | 0.002 |
