# Supplementary material for: Experimental Chagas disease-induced perturbations of the fecal microbiome and metabolome
Source: PLoS Negl Trop Dis. 2018 Mar 12;12(3):e0006344. doi: 10.1371/journal.pntd.0006344 (PMC5864088; doi:10.1371/journal.pntd.0006344)
Supplement: S1 Fig — (A) Weight change. *, p<0.05 (Mann-Whitney, FDR-corrected). (B) Mortality. (C) Luminescence. (D) Representative hematoxylin-eosin staining of colon segments from uninfected (top) and infected (bottom) mice. (DOCX) [file pntd.0006344.s006.docx]

**S1 Fig. Disease progression.** (**A**) Weight change**.** *, p<0.05 (Mann-Whitney, FDR-corrected). (**B**) Mortality. (**C**) Luminescence. (**D**) Representative hematoxylin-eosin staining of colon segments from uninfected (top) and infected (bottom) mice.

**B**

**A**


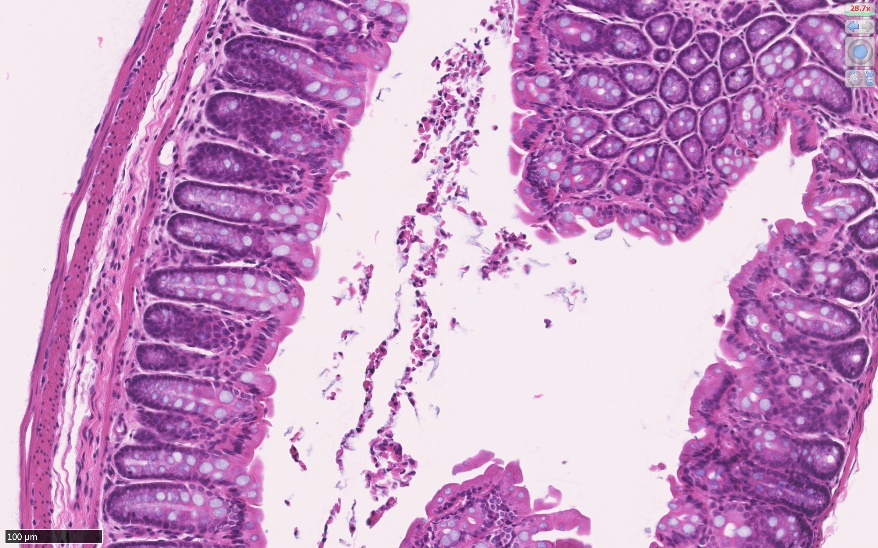

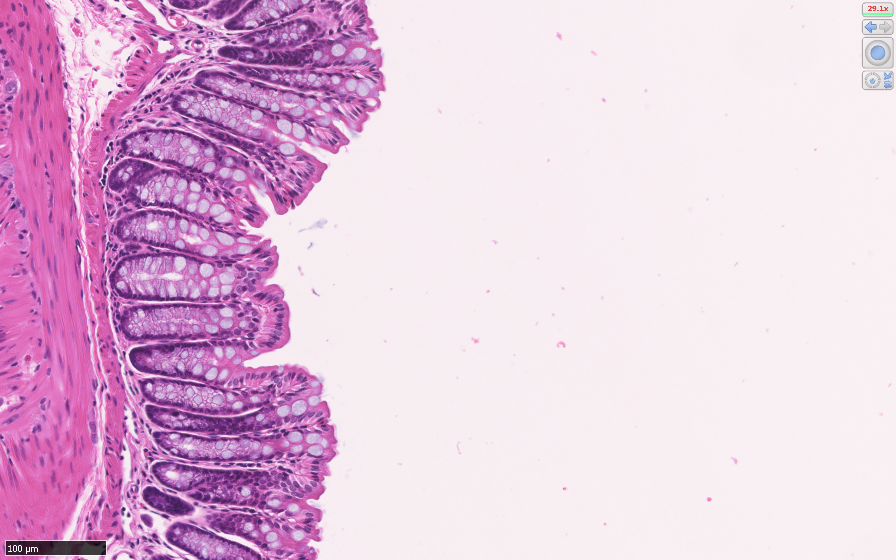

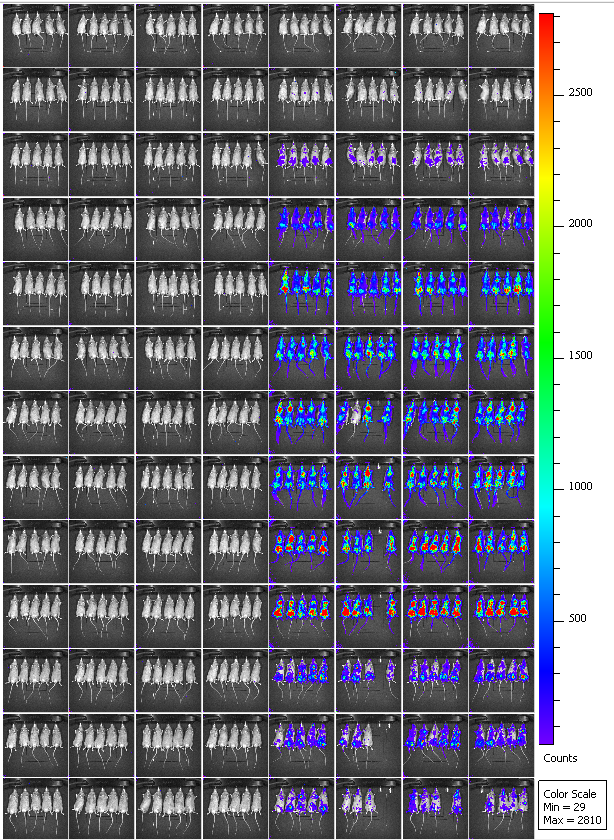

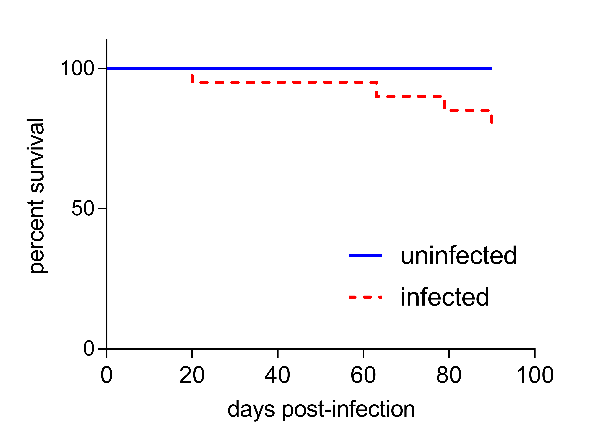

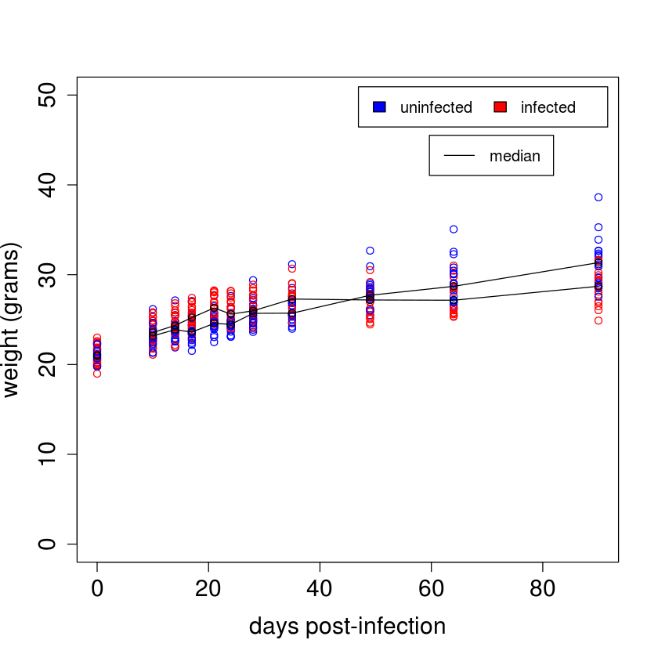


infected

uninfected

**D**

infected

uninfected

**C**

days post-infection

0

90

*

*
