# Supplementary material for: Experimental Chagas disease-induced perturbations of the fecal microbiome and metabolome
Source: PLoS Negl Trop Dis. 2018 Mar 12;12(3):e0006344. doi: 10.1371/journal.pntd.0006344 (PMC5864088; doi:10.1371/journal.pntd.0006344)
Supplement: S2 Fig — (A) Microbiome dataset (weighted UniFrac). (B) Metabolome dataset (Bray-Curtis-Faith). *, p<0.05 (Mann-Whitney, FDR-corrected). (DOCX) [file pntd.0006344.s007.docx]

**S2 Fig. Within-group and between-group distances.** (**A**) Microbiome dataset (weighted UniFrac). (**B**) Metabolome dataset (Bray-Curtis-Faith). *, p<0.05 (Mann-Whitney, FDR-corrected).


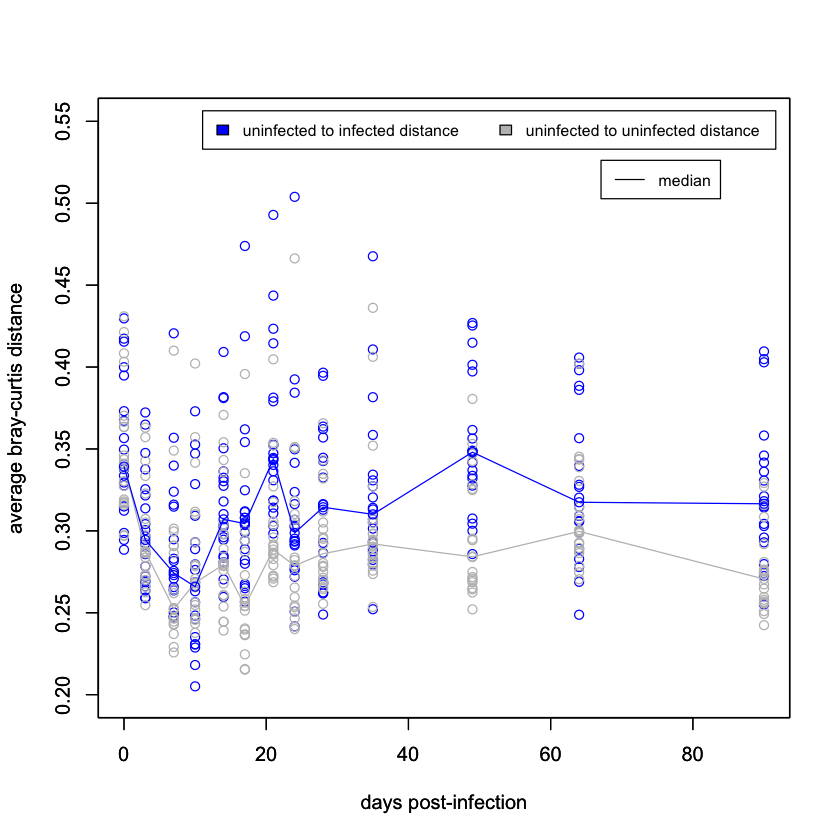

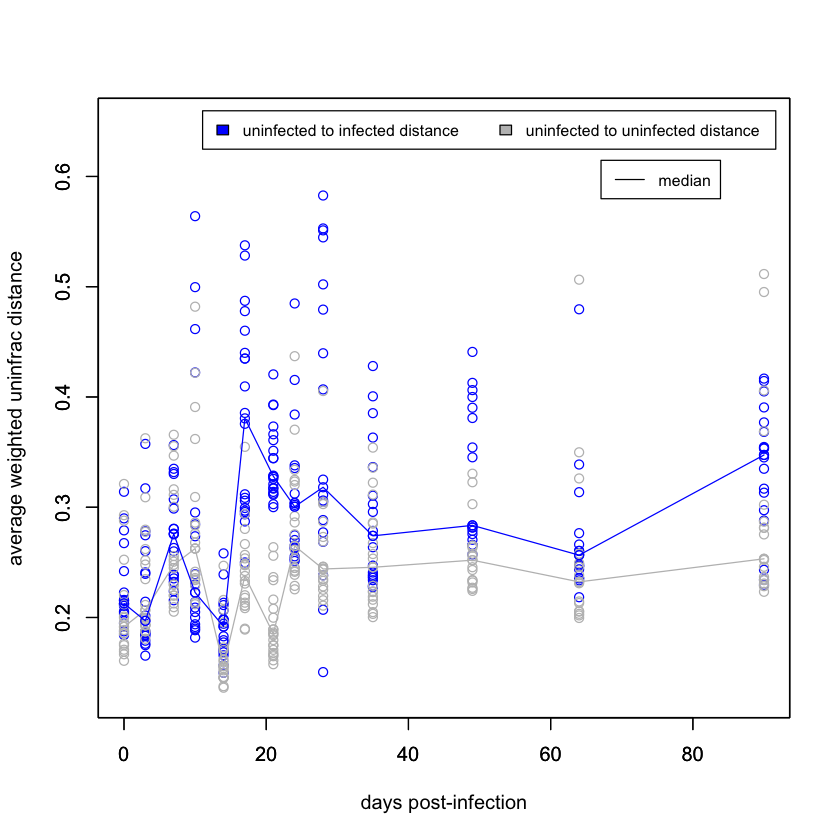


**B**

**A**

*

*

*

*

*

*

*

*

*

*

*
