# Supplementary material for: Experimental Chagas disease-induced perturbations of the fecal microbiome and metabolome
Source: PLoS Negl Trop Dis. 2018 Mar 12;12(3):e0006344. doi: 10.1371/journal.pntd.0006344 (PMC5864088; doi:10.1371/journal.pntd.0006344)
Supplement: S5 Fig — Values in parentheses indicate Student asymptotic p-value for the correlation. (DOCX) [file pntd.0006344.s010.docx]

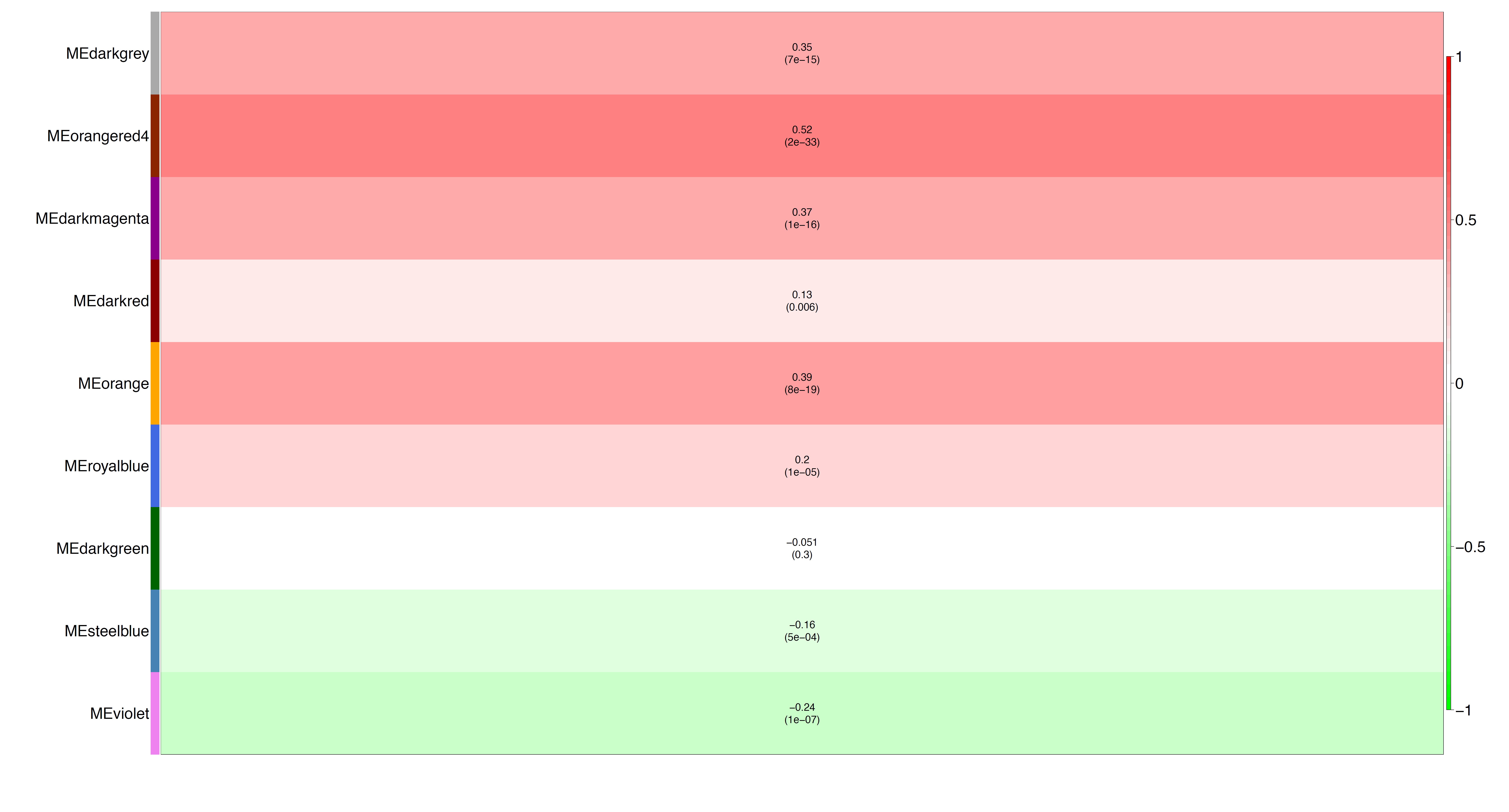
**S5 Fig. Parasite burden-associated microbial and metabolite module correlation.** Values in parentheses indicate Student asymptotic p-value for the correlation.
