# Supplementary material for: Experimental Chagas disease-induced perturbations of the fecal microbiome and metabolome
Source: PLoS Negl Trop Dis. 2018 Mar 12;12(3):e0006344. doi: 10.1371/journal.pntd.0006344 (PMC5864088; doi:10.1371/journal.pntd.0006344)
Supplement: S6 Fig — (A) LA/CLA Molecular network. (B) CLA isomers. Extracted ion chromatogram for m/z 281.200–281.260 (green). Numbers indicate CLA isomers with similar MS/MS fragmentation and arrow indicates sample peak matched to linoleic acid/conjugated linoleic acid authentic standard (red/blue). (C) Mirror plot showing spectral match of experimental spectrum (top, black) to library reference for CLA (bottom, green). (D) Overall comparable levels of LA/CLA between infected and uninfected samples. *, p<0.01 (Mann-Whitney, FDR-corrected). (DOCX) [file pntd.0006344.s011.docx]

**S6 Fig. Linoleic acid/conjugated linoleic acid identification.** (**A**) LA/CLA molecular network. (**B**) CLA isomers. Extracted ion chromatogram for *m/z* 281.200-281.260 (green). Numbers indicate CLA isomers with similar MS/MS fragmentation and arrow indicates sample peak matched to linoleic acid/conjugated linoleic acid authentic standard (red/blue). (**C**) Mirror plot showing spectral match of experimental spectrum (top, black) to library reference for CLA (bottom, green). (**D**) Overall comparable levels of LA/CLA between infected and uninfected samples. *, p<0.01 (Mann-Whitney, FDR-corrected).


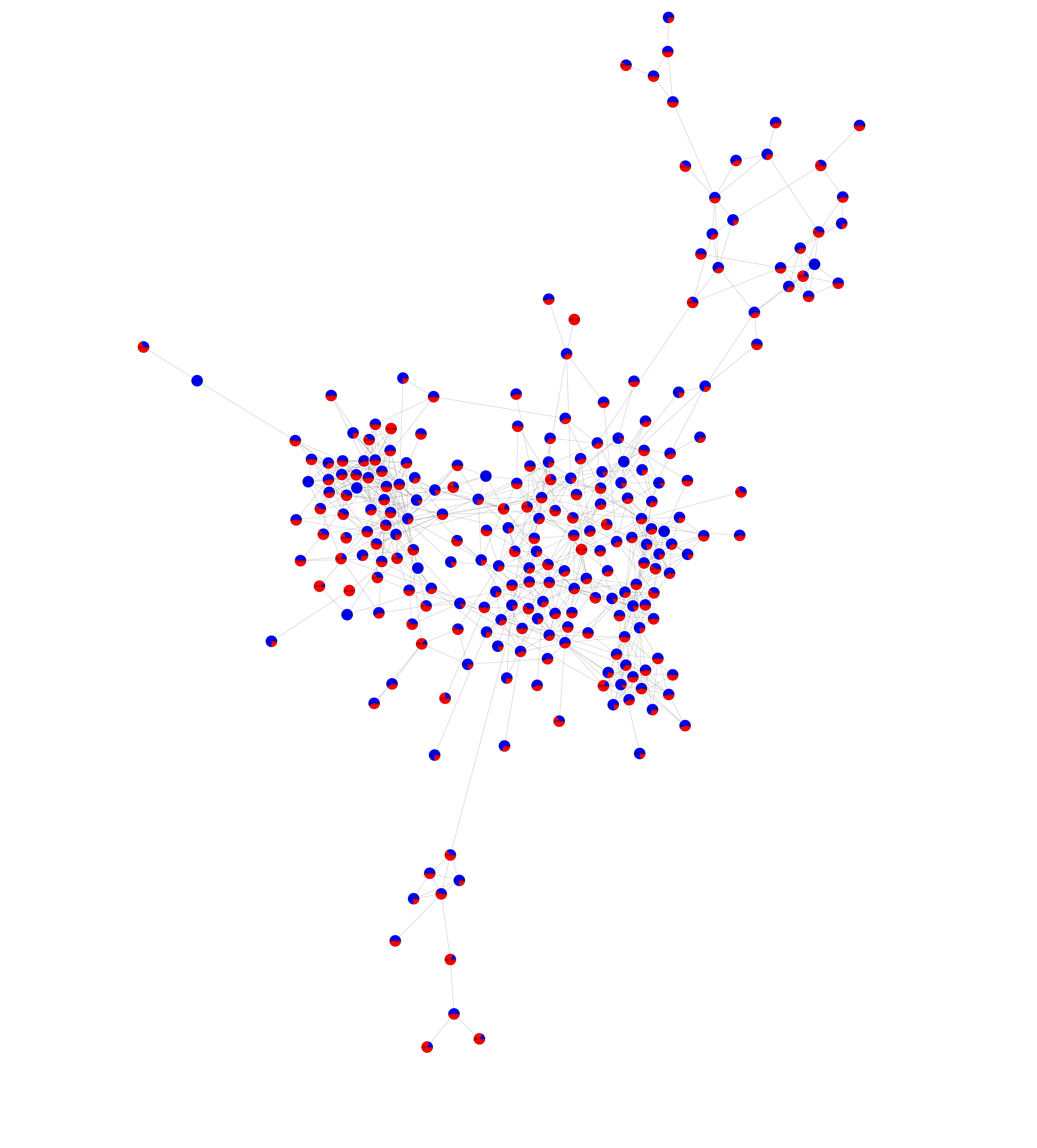


**A**

**B**


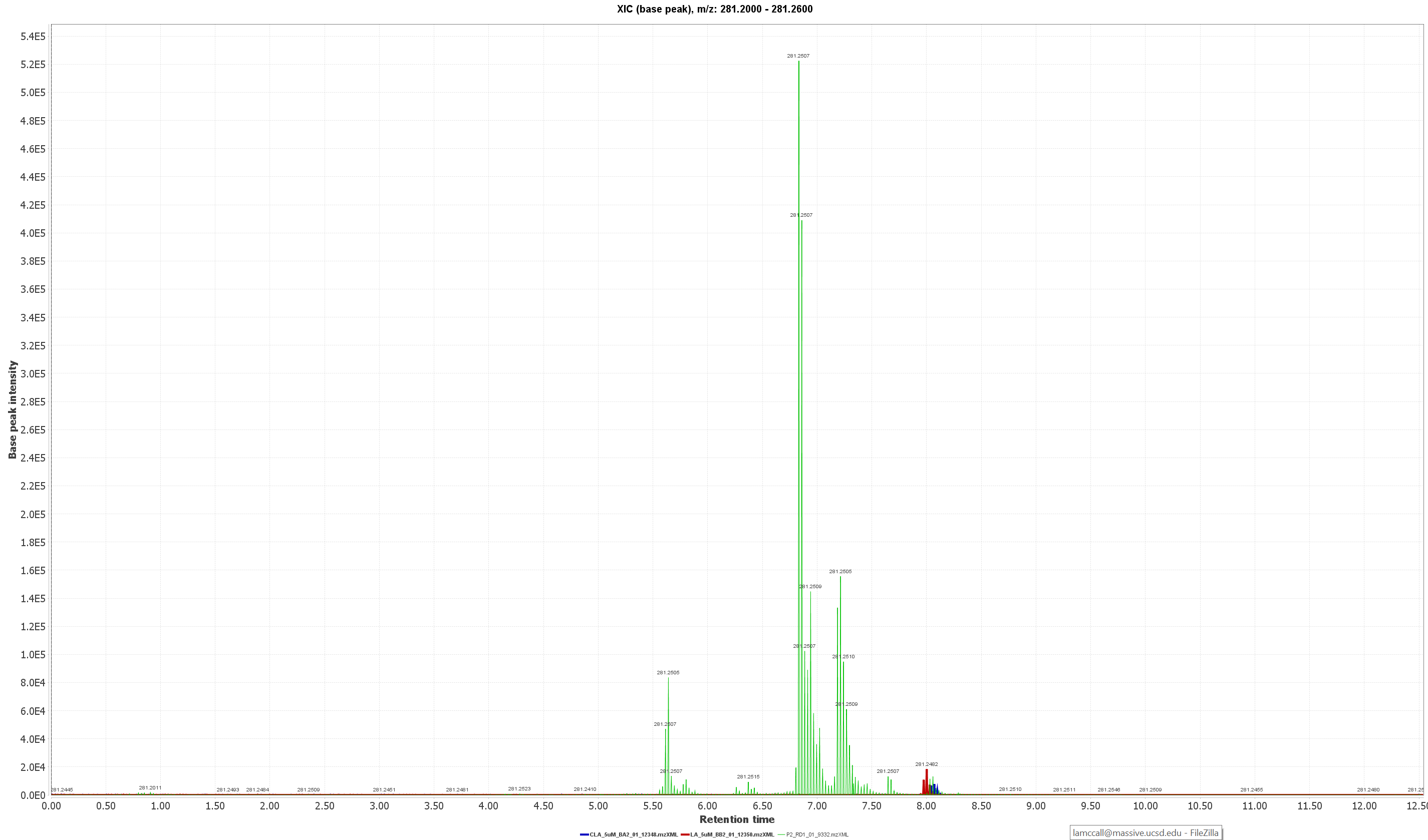


**1**

**2**

**4**

**3**

**5**

LA/CLA matches

positively correlated with burden

uninfected

infected

**D**

*


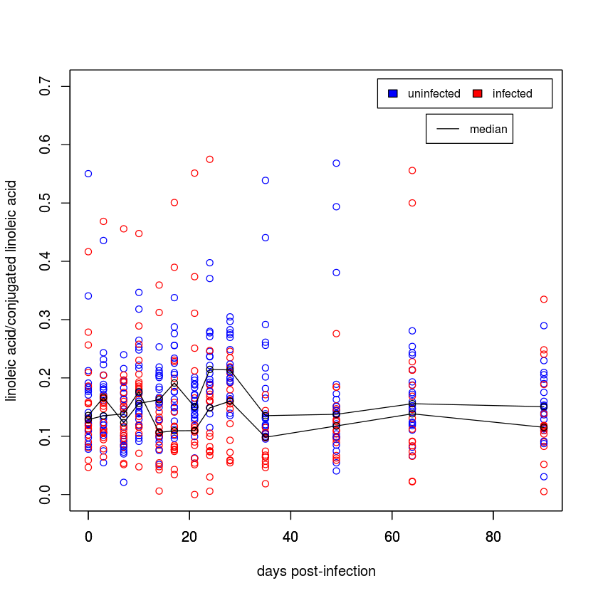


*

*

*


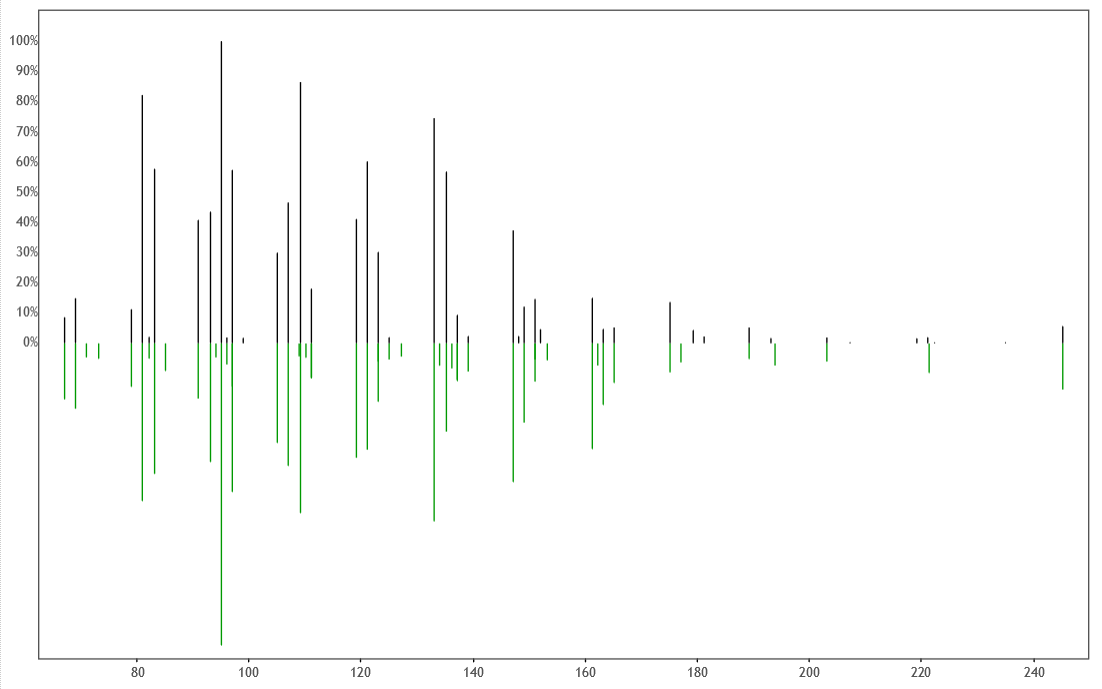


**C**
