# Supplementary material for: Experimental Chagas disease-induced perturbations of the fecal microbiome and metabolome
Source: PLoS Negl Trop Dis. 2018 Mar 12;12(3):e0006344. doi: 10.1371/journal.pntd.0006344 (PMC5864088; doi:10.1371/journal.pntd.0006344)
Supplement: S7 Fig — (A) m/z 263.241 RT 422s. (B) m/z 281.250 RT 355s. (C) m/z 281.251 RT 336s. (D) m/z 281.251 RT 383s. (E) m/z 283.266 RT 435s. (F) m/z 299.261 RT 336s. (G) m/z 313.276 RT 402s. (H) m/z 317.271 RT 336s. (I) m/z 331.287 RT 374s. (J) m/z 331.287 RT 384s. (K) m/z 365.234 RT 384s. *, p<0.05 (Mann-Whitney, FDR-corrected). (DOCX) [file pntd.0006344.s012.docx]

**S7 Fig. Co-modulated LA/CLA derivatives.** (**A**) *m/z* 263.241 RT 422s. (**B**) *m/z* 281.250 RT 355s. (**C**) *m/z* 281.251 RT 336s. (**D**) *m/z* 281.251 RT 383s. (**E**) *m/z* 283.266 RT 435s. (**F**) *m/z* 299.261 RT 336s. (**G**) *m/z* 313.276 RT 402s. (**H**) *m/z* 317.271 RT 336s. (**I**) *m/z* 331.287 RT 374s. (**J**) *m/z* 331.287 RT 384s. (**K**) *m/z* 365.234 RT 384s. *, p<0.05 (Mann-Whitney, FDR-corrected).

**C**

**A**

**B**


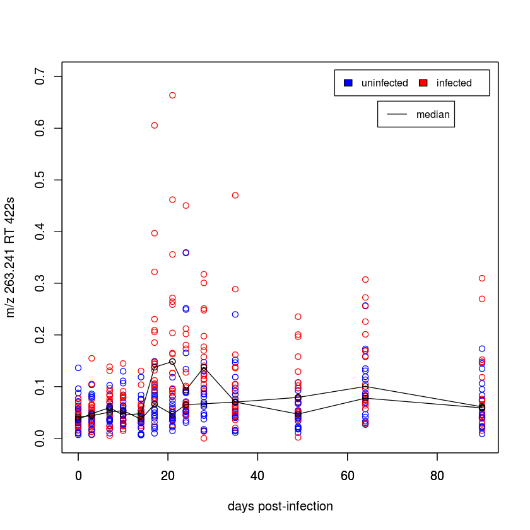


*

*

*

*


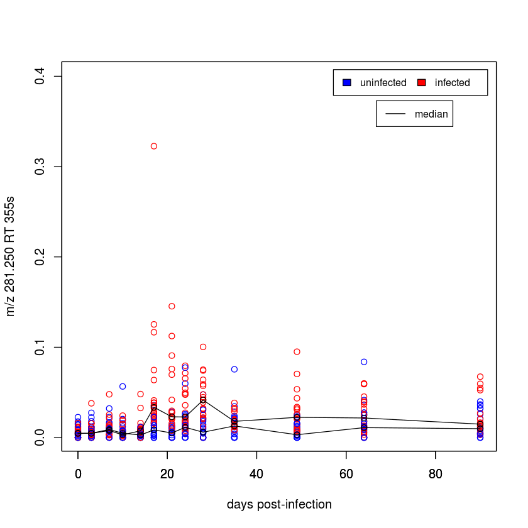


*

*

*

*

*

*


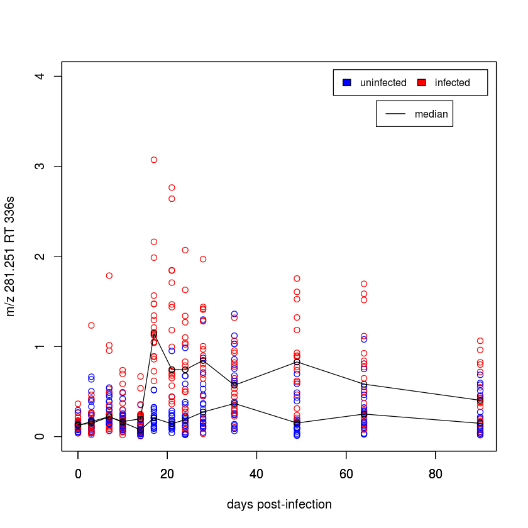


*

*

*

*

*

*

*


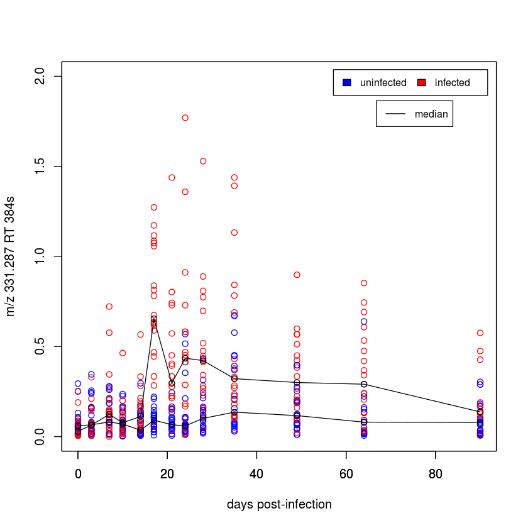


*

*

*

*

*

*

*


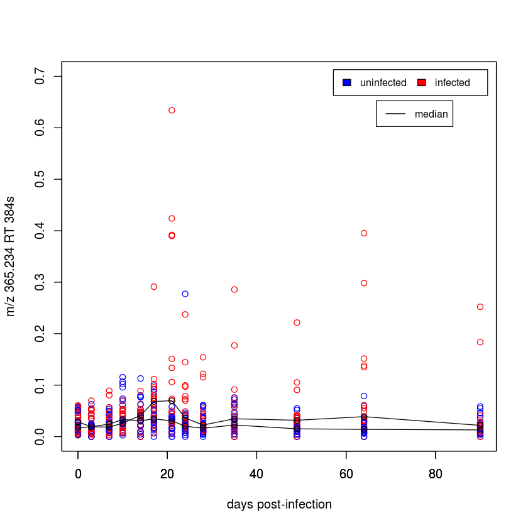


*

*

*

*

**J**

**K**

**I**

**H**


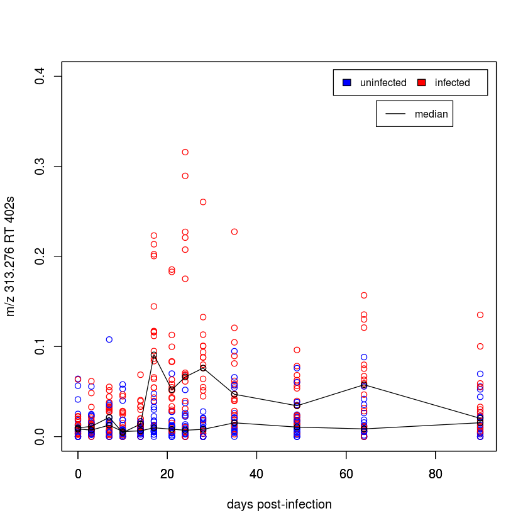

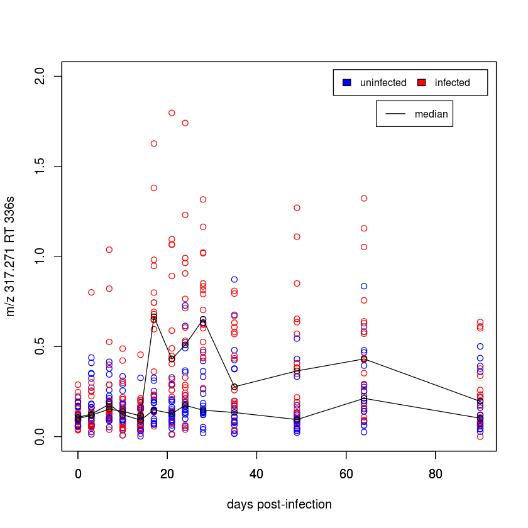

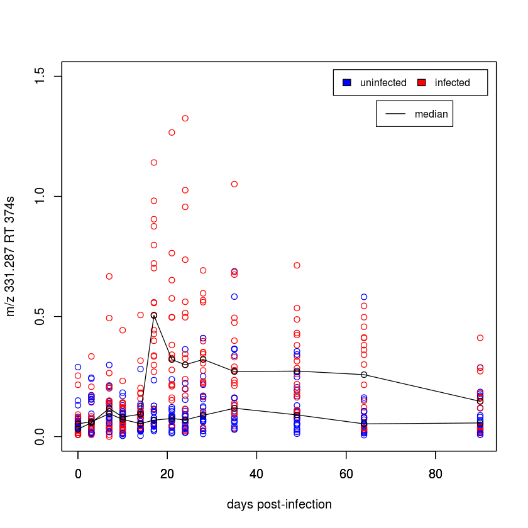


*

*

*

*

*

*

*

*

*

*

*

*

*

*

*

*

*

*

*

**G**


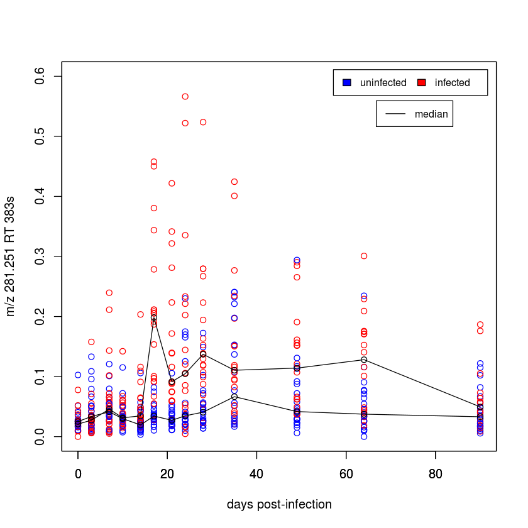

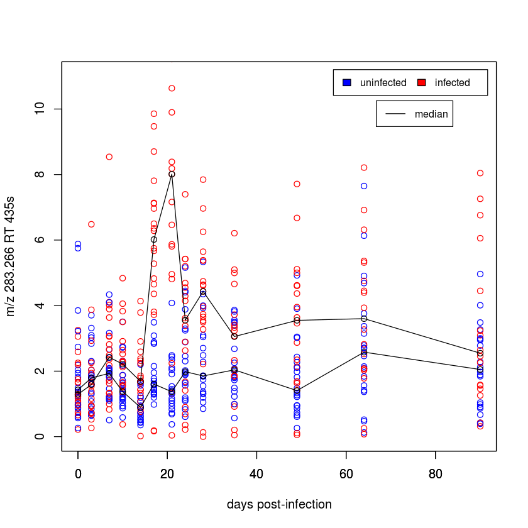

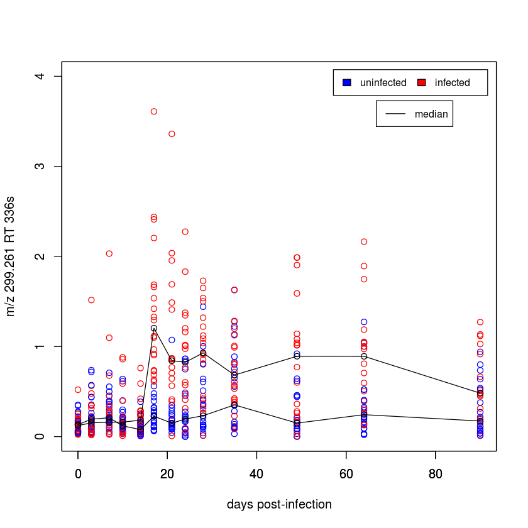


*

*

*

*

*

*

*

*

*

*

*

*

*

*

*

*

*

*

*

**F**

**E**

**D**
