# Supplementary material for: Experimental Chagas disease-induced perturbations of the fecal microbiome and metabolome
Source: PLoS Negl Trop Dis. 2018 Mar 12;12(3):e0006344. doi: 10.1371/journal.pntd.0006344 (PMC5864088; doi:10.1371/journal.pntd.0006344)
Supplement: S8 Fig — (A) Mirror plot showing spectral match of experimental spectrum (top) to cholic acid [M+H-3H2O] library reference (bottom). (B) Cholic acid molecular network. (C) Comparable cholic acid levels in infected and uninfected mice. (D) m/z 357.281 RT 337s. (E) m/z 357.281 RT 371s. (F) m/z 358.285 RT 371s. (G) m/z 358.285 RT 387s. (H) m/z 375.291 RT 393s. *, p<0.05 (Mann-Whitney, FDR-corrected). (DOCX) [file pntd.0006344.s013.docx]

**S8 Fig. Co-modulated cholic acid derivatives.** (**A**) Mirror plot showing spectral match of experimental spectrum (top) to cholic acid [M+H-3H_2_O] library reference (bottom). (**B**) Cholic acid molecular network. (**C**) Comparable cholic acid levels in infected and uninfected mice. (**D**) *m/z* 357.281 RT 337s. (**E**) *m/z* 357.281 RT 371s. (**F**) *m/z* 358.285 RT 371s. (**G**) *m/z* 358.285 RT 387s. (**H**) *m/z* 375.291 RT 393s. *, p<0.05 (Mann-Whitney, FDR-corrected).


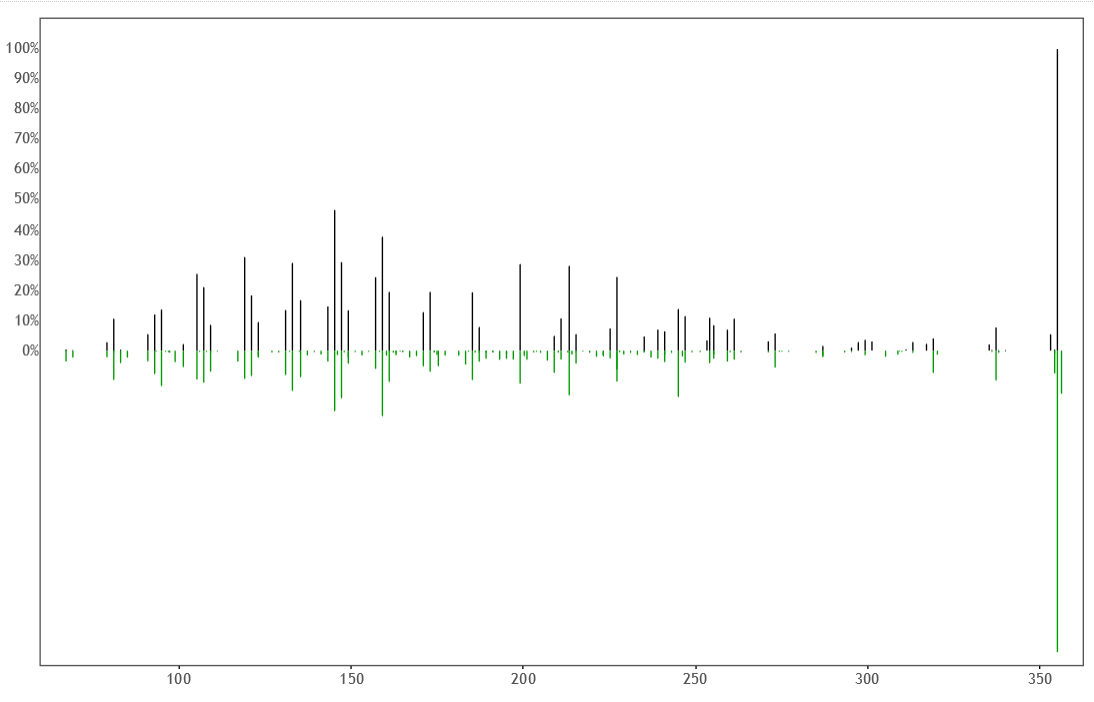


**A**


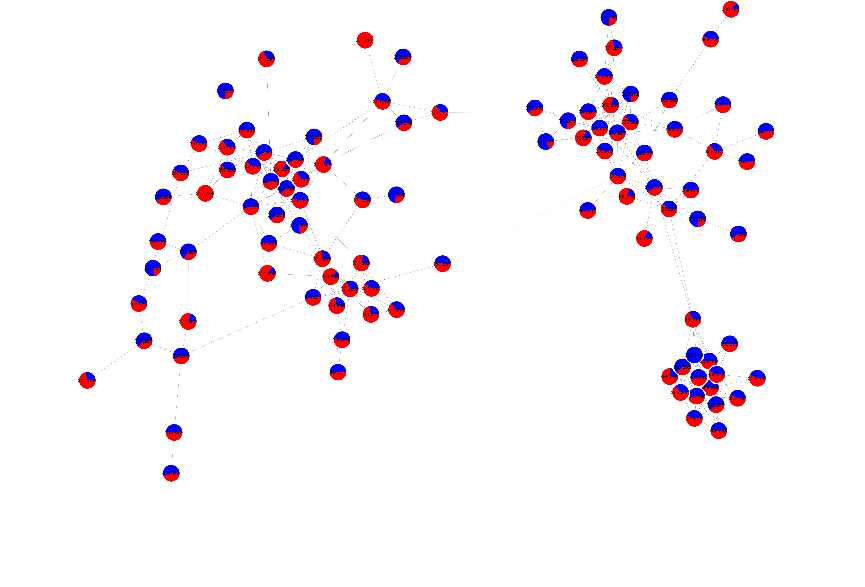


uninfected

infected


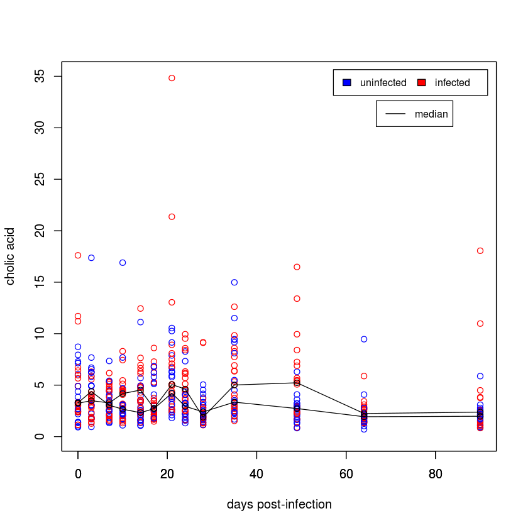


**C**

*


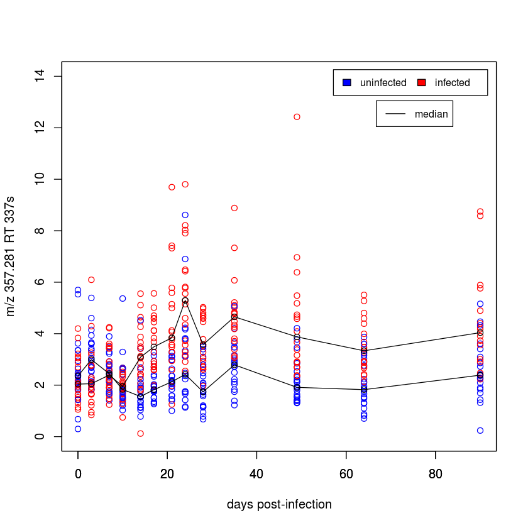

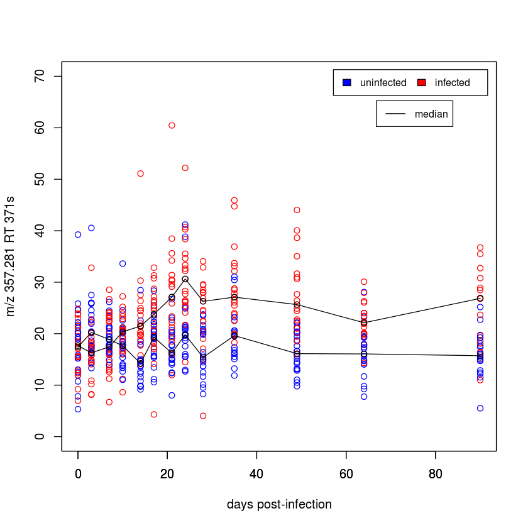


*

*

**D**

**E**

*

*

*

*

*

*

*

*

*

*

*

*

*

*

*

*

*

*


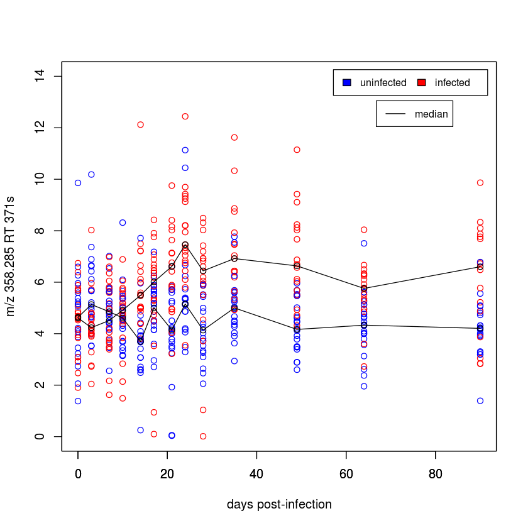


*

**F**

*

*

*

*

*

*

*

*


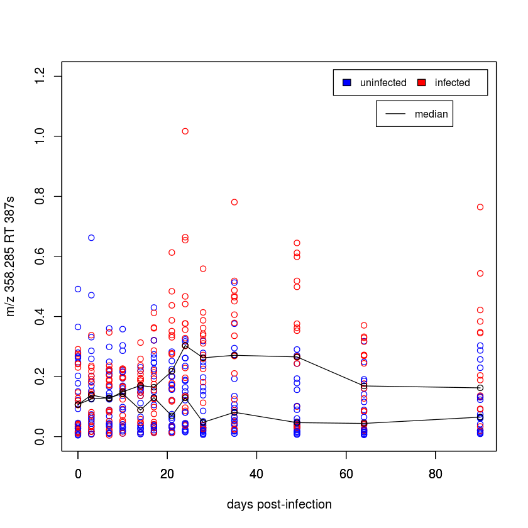

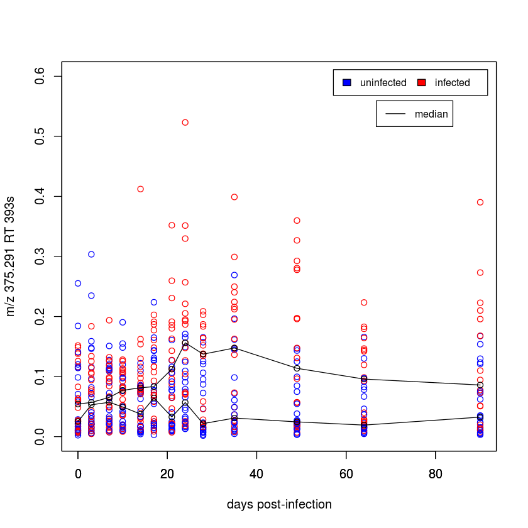


**G**

**H**

*

*

*

*

*

*

*

*

*

*

*

*

*

*

*

**B**
