# Supplementary material for: Experimental Chagas disease-induced perturbations of the fecal microbiome and metabolome
Source: PLoS Negl Trop Dis. 2018 Mar 12;12(3):e0006344. doi: 10.1371/journal.pntd.0006344 (PMC5864088; doi:10.1371/journal.pntd.0006344)
Supplement: S9 Fig — (A) Phylum level. (B) Class level. (C) Order level. (D) Family level. Each bar represents a given mouse. (DOCX) [file pntd.0006344.s014.docx]

**S9 Fig. Pre-infection fecal microbiome composition.** (**A**) Phylum level. (**B**) Class level. (**C**) Order level. (**D**) Family level. Each bar represents a given mouse.

**A**

**B**

**C**

**D**
