# Supplementary material for: Experimental Chagas disease-induced perturbations of the fecal microbiome and metabolome
Source: PLoS Negl Trop Dis. 2018 Mar 12;12(3):e0006344. doi: 10.1371/journal.pntd.0006344 (PMC5864088; doi:10.1371/journal.pntd.0006344)
Supplement: S10 Fig — Various views and principal coordinates of PCoA plots with samples, blanks and pooled QC samples (A) and with only samples and pooled QC (B). Blank samples are distinctly different from all other samples, while pooled QC are in the middle of the PCoA. Distinct clustering of high parasite burden samples is highlighted with dotted oval, where permitted by the viewing angle. (DOCX) [file pntd.0006344.s015.docx]

**S10 Fig. Principal coordinates analysis of metabolomics samples and controls.** Various views and principal coordinates of PCoA plots with samples, blanks and pooled QC samples (**A**) and with only samples and pooled QC (**B**). Blank samples are distinctly different from all other samples, while pooled QC are in the middle of the PCoA. Distinct clustering of high parasite burden samples is highlighted with dotted oval, where permitted by the viewing angle.


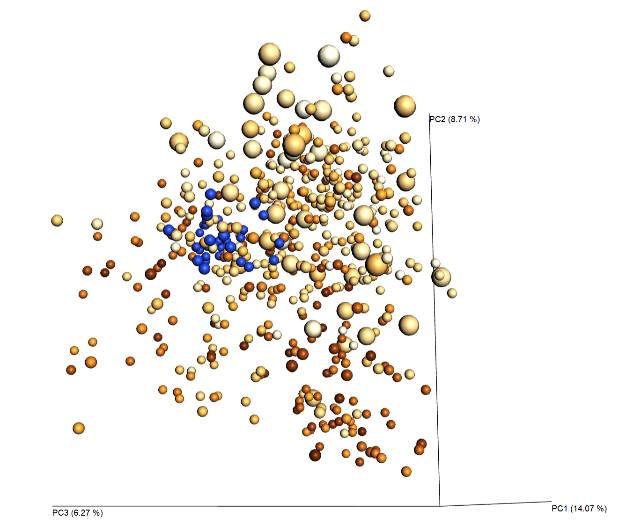

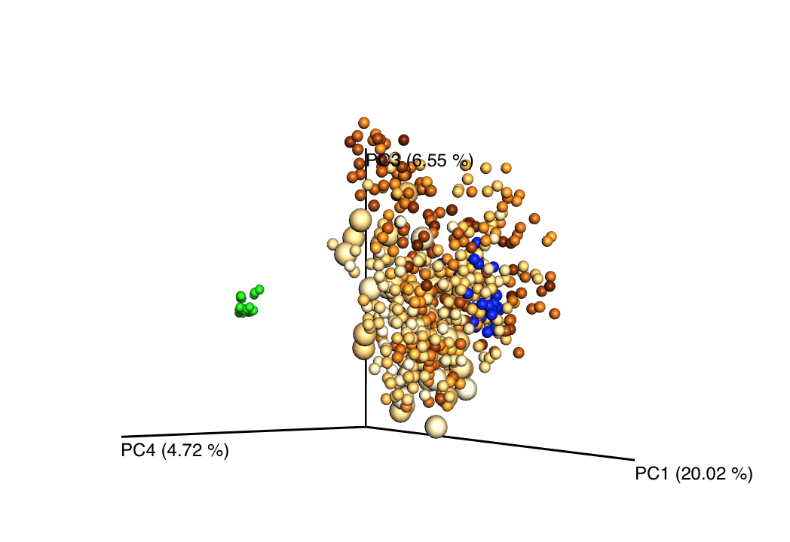

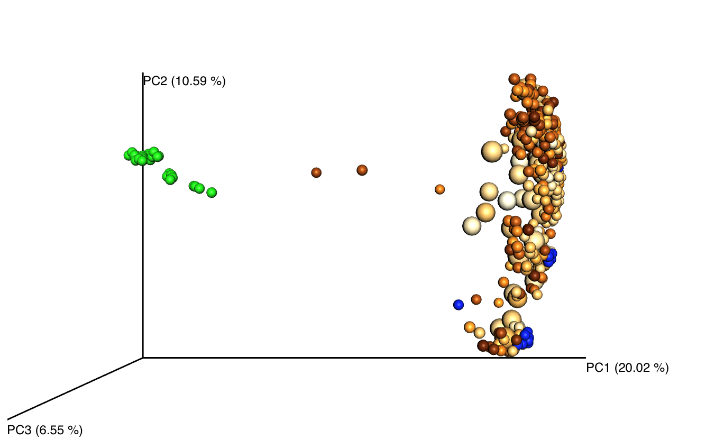

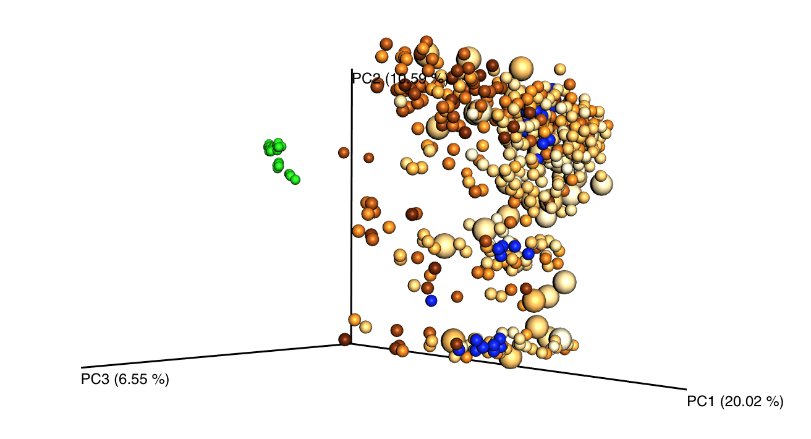


intestinal parasite burden

day 0

days 3-90


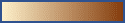


pooled QC

blank

**A**

**B**
